# The Antimicrobial Peptide γ-Thionin from Habanero Chile (*Capsicum chinense*) Induces Caspase-Independent Apoptosis on Human K562 Chronic Myeloid Leukemia Cells and Regulates Epigenetic Marks

**DOI:** 10.3390/molecules28093661

**Published:** 2023-04-23

**Authors:** Luis José Flores-Alvarez, Paola Jiménez-Alcántar, Alejandra Ochoa-Zarzosa, Joel E. López-Meza

**Affiliations:** Centro Multidisciplinario de Estudios en Biotecnología, Facultad de Medicina Veterinaria y Zootecnia, Universidad Michoacana de San Nicolás de Hidalgo, Km 9.5 Carretera Morelia-Zinapécuaro, Posta Veterinaria, Morelia C.P. 58893, Mexico; ljfa21@yahoo.com.mx (L.J.F.-A.); 0526707c@umich.mx (P.J.-A.); ochoaz@umich.mx (A.O.-Z.)

**Keywords:** leukemia, calpains, apoptosis, antimicrobial peptides, γ-thionin, epigenetic regulation

## Abstract

Cancer is a relevant health problem worldwide. In 2020, leukemias represented the 13th most commonly reported cancer cases worldwide but the 10th most likely to cause deaths. There has been a progressive increase in the efficacy of treatments for leukemias; however, these still generate important side effects, so it is imperative to search for new alternatives. Defensins are a group of antimicrobial peptides with activity against cancer cells. However, the cytotoxic mechanism of these peptides has been described mainly for animal defensins. This study shows that defensin γ-thionin (*Capsicum chinense*) is cytotoxic to the K562 leukemia cells with an IC_50_ = 290 μg/mL (50.26 μM) but not for human peripheral blood mononuclear cells. Results showed that γ-thionin did not affect the membrane potential; however, the peptide modified the mitochondrial membrane potential (ΔΨm) and the intracellular calcium release. In addition, γ-thionin induced apoptosis in K562 cells, but the activation of caspases 8 and 9 was not detected. Moreover, the activation of calpains was detected at one hour of treatment, suggesting that γ-thionin activates the caspase-independent apoptosis. Furthermore, the γ-thionin induced epigenetic modifications on histone 3 in K562 cells, increased global acetylation (~2-fold), and specific acetylation marks at lysine 9 (H3K9Ac) (~1.5-fold). In addition, γ-thionin increased the lysine 9 methylation (H3K9me) and dimethylation marks (H3K9me2) (~2-fold), as well as the trimethylation mark (H3K9me3) (~2-fold). To our knowledge, this is the first report of a defensin that triggers caspase-independent apoptosis in cancer cells via calpains and regulating chromatin remodelation, a novel property for a plant defensin.

## 1. Introduction

Antimicrobial peptides (AMPs) are small molecules (12–100 aa), mainly amphipathic and cationic, produced by a wide range of organisms [1,2]. AMPs are mostly antimicrobial molecules. However, they also regulate the immune response, wound repair, angiogenesis induction, and cytotoxicity [2,3]. Today, more than 3000 AMPs have been reported, and ~7% showed anticancer activity [4]; nevertheless, the cytotoxic mechanisms have not been described in detail [2,5,6,7].

AMPs from plants (PAP) possess 40–50 aa residues (3–10 kDa), are rich in cysteine, and can be expressed constitutively or induced by damage [8]. PAP are grouped into several families, but only three (thionins, defensins, and cyclotides) show cytotoxic activity [4,8,9].

The plant defensins (PDs) comprise a group of PAP whose structure is composed of helix-α and β-sheet, resembling the animal and insect defensins [10,11]. The PDs are present in seeds, leaves, tubers, fruits, roots, and barks [12]. They have antibacterial, antifungal, and insecticide activities, in addition to influencing plant growth and development [8]. The anticancer potential of PDs lies in their selectivity against cancer cells from different origins (breast, colon, prostate, leukemia) [9]. The cytotoxicity of these PDs involves the activation of apoptosis or necrosis. For example, NaD1 (*Nicotiana alata*) and TPP3 (tomato) defensins favor necrosis in Jurkat and U937 cells through the peptide binding to the phosphatidylinositol 4,5-bisphosphate [13,14]. On the other hand, the cytotoxicity of PaDef defensin (*Persea americana* var. drymifolia) on K562 chronic myeloid cells and MCF-7 cells involves extrinsic and intrinsic apoptosis, respectively [15,16].

Apoptosis determines cell death in a regulated manner in response to physiological or pathological stimuli [17]. Extrinsic stimuli can mediate apoptosis through death surface receptors, such as TNF-α (tumor necrosis factor-α), Fas (CD95/APO1), and TRAIL (TNF-related apoptosis-inducing ligand) or by intrinsic stimulation mediated by mitochondria [17,18]. However, apoptosis can also be activated by a caspase-independent pathway, which could engage calpains. Calpains are cysteine proteases located in the cytoplasm and mitochondria. They are calcium-dependent and trigger caspases-independent apoptosis due to their interaction with pro-apoptotic proteins, such as Bax, mediated by AIF (Apoptosis Induction Factor) [19,20,21]. In this sense, the human cathelicidin LL-37 induces apoptosis caspases-independent in Jurkat [22] and colon cancer cells [23].

Cancer is one of the biggest causes of death worldwide. Leukemias represent the 13th most commonly reported cancer cases worldwide but the 10th most likely cancer to cause death in 2020, and can be classified by both the speed of evolution and the types of cells involved in acute or chronic leukemia (GLOBOCAN 2022). Chronic myeloid leukemia occurs mostly in adults and is less frequent in children. In recent decades, there has been a progressive increase in the efficacy of treatments for leukemias, increasing patients’ survival rates. However, treatments are often associated with a series of toxic or side effects that negatively influence patient’s quality of life. In this sense, searching for molecules that activate apoptosis in cancer cells and whose effects are related to modifications in chromatin remodeling could be an anticancer strategy that prevents the development of drug resistance or reverses the epigenetic alterations responsible for developing resistance to treatments.

Previously, we reported that the PaDef defensin is cytotoxic for chronic myeloid cell line K562 [16]. Moreover, PaDef is cytotoxic to acute lymphoid leukemia cell line Jurkat, and this activity was associated with epigenetic modifications [24]. In addition, we reported that γ-thionin, a defensin (50 aa) from *Capsicum chinense*, has antimicrobial and cytotoxic activity against different cancer cell lines [25]. γ-thionin was cytotoxic to MCF-7 cells in a concentration-dependent manner, with an IC_50_ = 117.29 μg/mL. Additionally, this AMP induced apoptosis in these cells, and did not affect the membrane integrity or cytosolic calcium [26]. Here, we demonstrated that γ-thionin is cytotoxic toward K562 cells by inducing apoptosis, but it did not affect peripheral blood mononuclear cells (PBMC). The mechanism activated was caspase-independent apoptosis mediated by calpains. In addition, γ-thionin regulates epigenetic marks in this cell line.

## 2. Results

### 2.1. γ-Thionin Defensin Is Cytotoxic to K562 Cells but Not to PBMC

**Figure 1:**
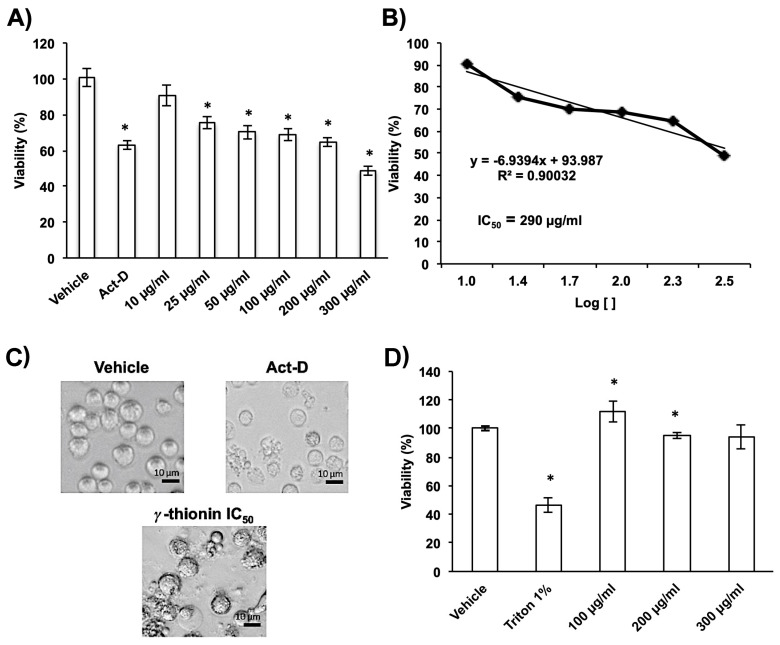
γ-Thionin is cytotoxic to K562 leukemia cells. (**A**) K562 cells were treated with γ-thionin (10, 25, 50, 100, 200, and 300 µg/mL), and viability was evaluated by MTT assay at 24 h. Cell viability is shown as the percentage of viable cells concerning cells treated with vehicle (DMSO 1.2%). Actinomycin D (Act-D, 80 μg/mL) was used as a positive control of death. Data represent the mean of three independent experiments performed in triplicate. * Indicates statistically significant differences concerning vehicle (*p* < 0.05). (**B**) The half maximal inhibitory concentration (IC_50_) of γ-thionin on K562 cells was calculated by regression analysis; IC_50_ = 290 μg/mL. (**C**) K562 cell morphology after different treatments. Representative photographs taken by light field microscopy are shown. Scale bars: 10 μm. (**D**) Effect of γ-thionin on the viability of human peripheral blood mononuclear cells. Cells were treated with γ-thionin (100, 200, and 300 μg/mL), vehicle (DMSO 1.2%), and triton 1% as a positive control of death. Viability was evaluated by MTT assays at 24 h. Data represent the average of three independent experiments performed in triplicate. Data were analyzed by *t*-student concerning to vehicle. * Indicates statistically significant differences concerning vehicle (*p* < 0.05).

We evaluated the cytotoxicity of γ-thionin on K562 leukemia cells and PBMC by MTT assay using different concentrations (10–300 μg/mL). Results showed that γ-thionin was cytotoxic to K562 cells in a concentration-dependent manner (Figure 1A), with an IC_50_ = 290 µg/mL (50.26 µM) (Figure 1B,C). The IC_50_ was corroborated by flow cytometry using Syto 9. In addition, the morphological evaluation suggests that K562 cells showed alterations similar to apoptotic vesicles (Figure 1C). Likewise, we showed that γ-thionin did not affect PBMC viability (Figure 1D).

### 2.2. γ-Thionin Defensin Does Not Affect the Membrane Integrity of K562 Cells

**Figure 2:**
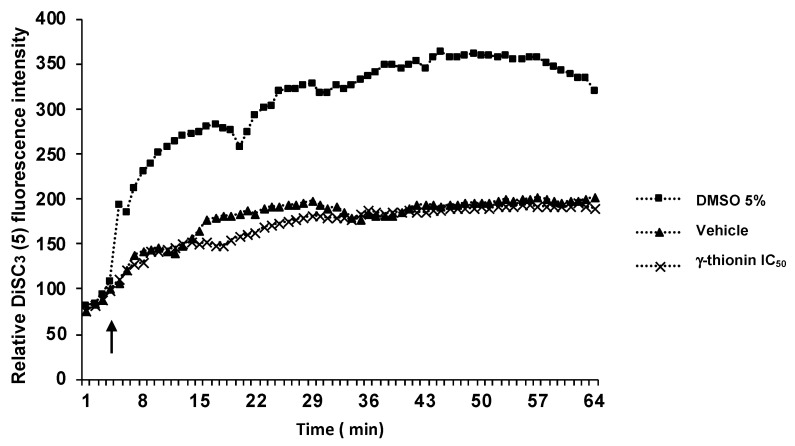
γ-thionin defensin does not affect the membrane of K562 cells. Changes in the membrane potential of K562 cells were measured using a membrane potential-sensitive dye. Cells were incubated with 200 μM DiSC3 (5) for 30 min at 37 °C and then treated with γ-thionin IC_50_, vehicle, and DMSO (5%, positive control). The assay was monitored for about 3.5 h with measurements every 2 min. Arrow indicates the time at which the treatments were added. γ-thionin = IC_50_ (290 µg/mL); vehicle = DMSO 1.2%.

To determine if the cytotoxicity of γ-thionin on K562 cells was related to cell membrane damage, we evaluated the plasma membrane potential using 3,3′-dipropylthiadicarbocyanine iodide (DiSC3 (5)). According to the results, γ-thionin did not affect membrane electrical potential compared with the positive control (DMSO 5%) (Figure 2).

### 2.3. γ-Thionin Defensin Induces Apoptosis without Activation of Caspases in K562 Cells but Induces Calpain Activation

**Figure 3:**
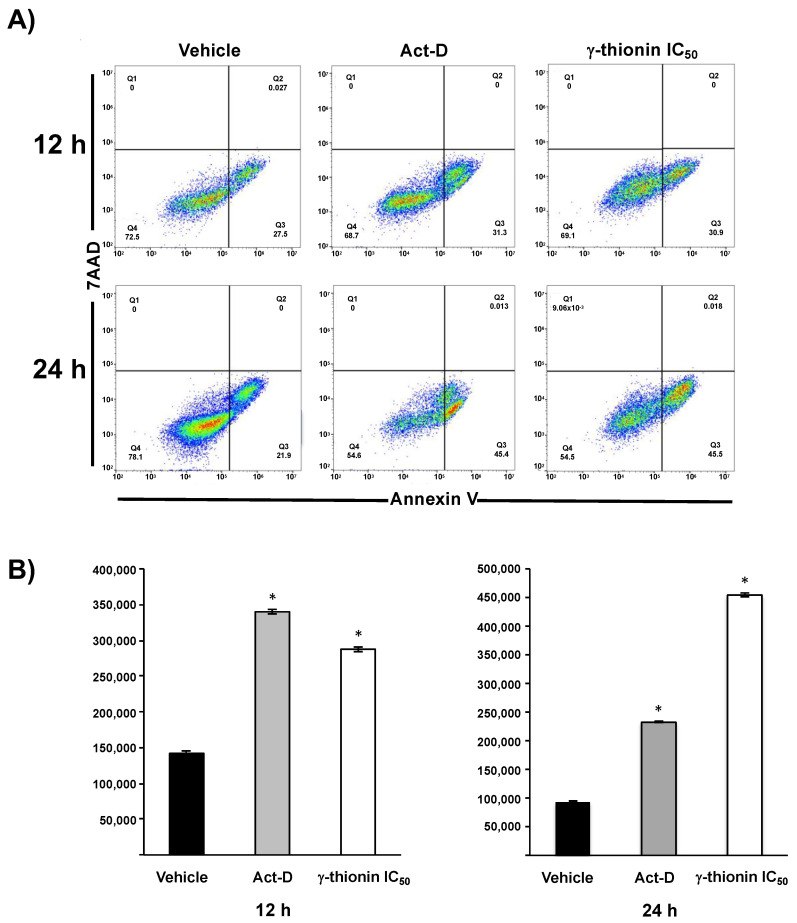
γ-thionin defensin induces apoptosis in K562 cells. (**A**) Cells were treated with γ-thionin IC_50_ (12 and 24 h) and analyzed by flow cytometry using Annexin V/7AAD staining. The Q4 quadrant indicates viable cells, the Q3 quadrant represents early apoptosis, the Q2 quadrant represents late apoptosis, and the Q1 quadrant contains necrotic cells. (**B**) Apoptosis analysis. The graphics show the relative fluorescence for each treatment time (relative units). Each bar shows the mean of triplicates ± SE of three independent experiments. A minimum of 10,000 events per sample were collected. Data were analyzed by *t*-student concerning to vehicle. * Indicates statistically significant differences concerning vehicle (*p* < 0.05). Act-D = 80 μg/mL; γ-thionin = IC_50_ (290 µg/mL); vehicle = DMSO 1.2%.

Different reports for AMPs have described apoptosis as the primary mechanism of activated cell death [15,27]. Therefore, we evaluated the apoptosis rate at 12 and 24 h. γ-Thionin induced early apoptosis since 12 h (>30%) in K562 cells (Figure 3A,B), which increased at 24 h (>45%) (Figure 3A,B); this effect was similar to that shown by actinomycin D (Figure 3) used as a positive control for apoptosis. In addition, we did not detect necrosis at times evaluated.

**Figure 4:**
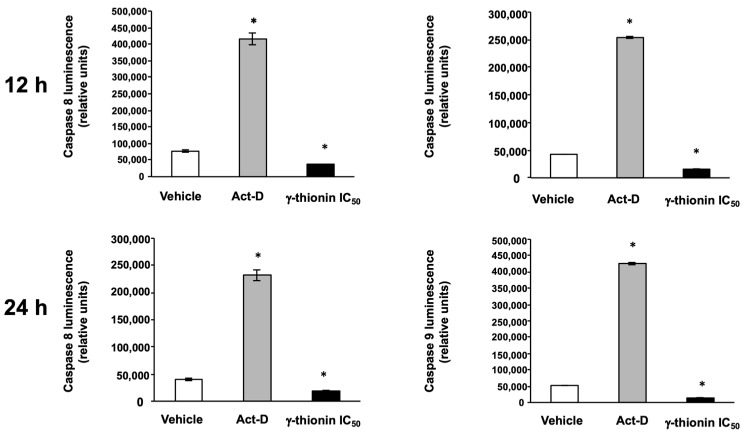
γ-thionin defensin does not induce the activity of caspases 8 and 9 in K562 cells. The activation of caspase 8 (Left graphs) and caspase 9 (Right graphs) was assessed in cells treated with γ-thionin IC_50_, vehicle, and Act-D (12 and 24 h). The activation of caspases was detected using Caspase-Glo 8 and 9 kits (Promega) according to the manufacturer’s instructions. Each bar shows the mean of triplicates ± SE of three independent experiments. Data were analyzed by *t*-student concerning to vehicle. * Indicates statistically significant differences concerning vehicle (*p* < 0.05). Act-D = 80 μg/mL; γ-thionin = IC_50_ (290 µg/mL); vehicle = DMSO 1.2%.

**Figure 5:**
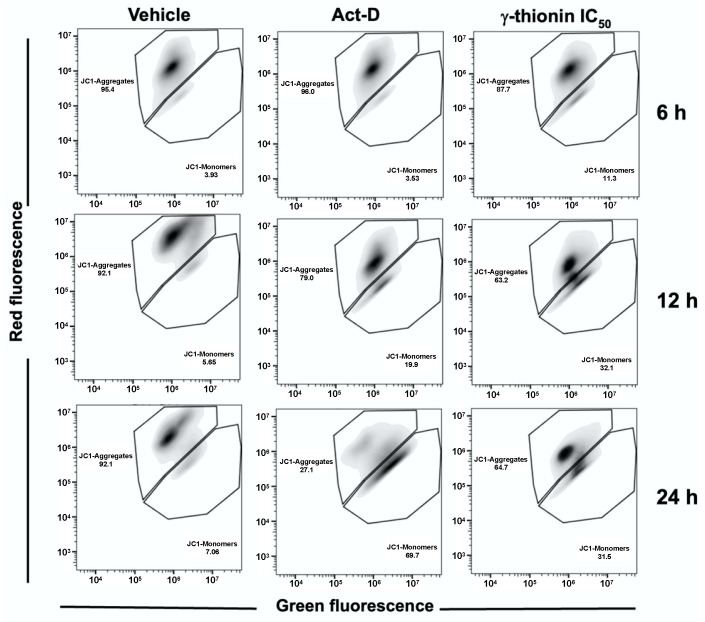
γ-thionin defensin modifies the mitochondrial membrane potential (ΔΨm) in K562 cells. Representative plots are shown. Cells were treated for 6, 12, or 24 h with vehicle, γ-thionin or Act-D. Cells were stained with JC-1 dye and fluorescence was measured by flow cytometry. Act-D = 80 μg/mL; γ-thionin = IC_50_ (290 µg/mL); vehicle = DMSO 1.2%.

**Figure 6:**
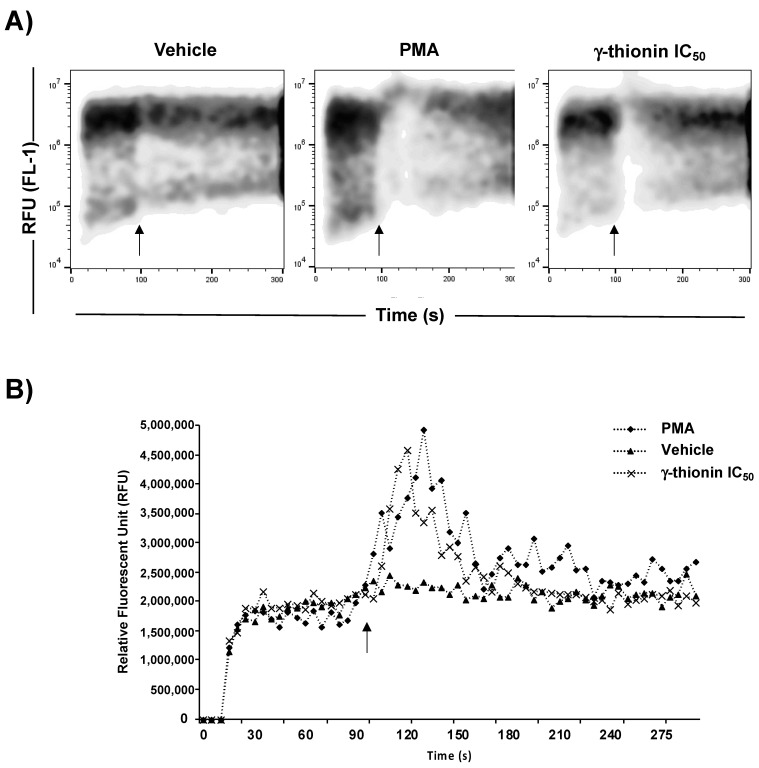
Intracellular calcium release in K562 cells is induced by γ-thionin defensin. The release of intracellular calcium was analyzed by flow cytometer using the Calcium Assay Kit (BD Biosciences). Measurements were performed for 5 min. For readings, a baseline fluorescence of 1 min was established, then the treatments were placed, and the fluorescence intensity was monitored for another 5 min. The panel shows representative plots (**A**) and relative fluorescence intensities for intracellular calcium release (**B**). Arrow indicates the time at which the treatments were added. PMA (3 μM) was used as a positive control; γ-thionin = IC_50_ (290 µg/mL); vehicle = DMSO 1.2%.

To identify the specific apoptotic pathway activated, we evaluated the activation of caspases 8 and 9. However, we did not detect activation of both caspases at 12 and 24 h (Figure 4), which were activated by actinomycin D. Moreover, γ-thionin modified the mitochondrial membrane potential ΔΨm since 6 h (Figure 5), reaching the level maximum at 12 h. Interestingly, γ-thionin induces intracellular calcium efflux at a similar level to PMA (Figure 6). Altogether, results suggest that γ-thionin activates a mechanism of caspase-independent apoptosis.

**Figure 7:**
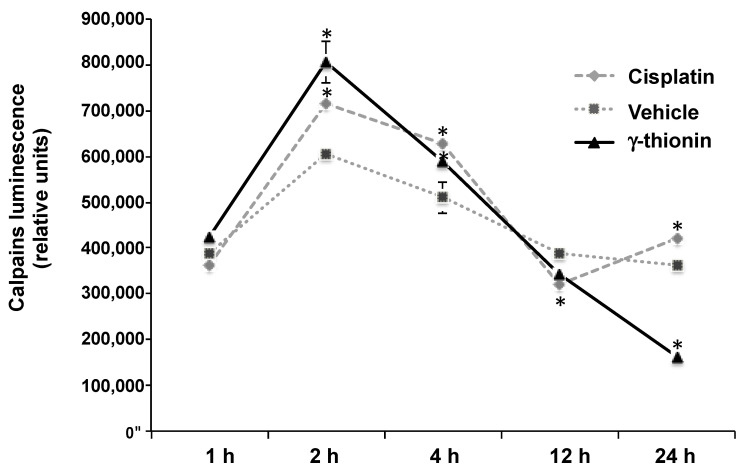
Activation of calpains in K562 cells treated with γ-thionin defensin. The induction of calpains was measured in cells treated with γ-thionin IC_50_ (1, 2, 4, 12, and 24 h) by luminescence. The activation of calpains was assessed with the Calpain-Glo kit (Promega) according to the manufacturer’s instructions. Each point shows the mean of replicates ± SE of three independent experiments. Data were analyzed by *t*-student concerning the vehicle. * Indicates statistically significant differences concerning vehicle (*p* < 0.05). Cisplatin (1 μg/mL) was used as a positive control of calpain activation. γ-thionin = IC_50_ (290 µg/mL); vehicle = DMSO 1.2%.

**Figure 8:**
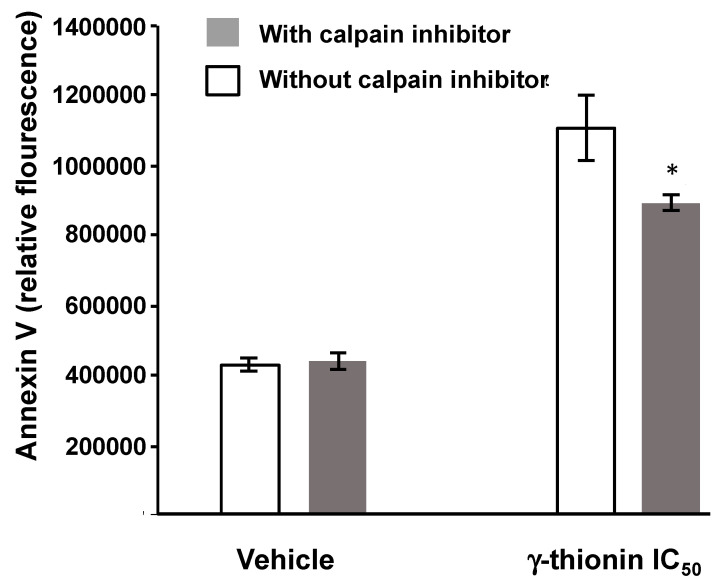
Participation of the calpains in the apoptosis activated by γ-thionin in K562 cells. Cells were treated for 1 h with the calpain-specific inhibitor N-Acetyl-Leu-Leu-norleucinal (5 mg/mL), and then cells were treated 24 h with vehicle or γ-thionin IC_50_. Apoptosis was assessed by flow cytometry measuring the relative fluorescence of Annexin V. Each bar shows the mean of triplicates ± SE of three independent experiments. Data were analyzed by a *t*-student. * Indicates statistically significant differences concerning γ-thionin treatment (*p* < 0.05). γ-thionin = IC_50_ (290 µg/mL); vehicle = DMSO 1.2%.

As the above results show, we analyzed whether γ-thionin induces calpain activity at different times. Results showed that this defensin favors the activation of calpains at 2 h (~25%) concerning the vehicle, which suggests that the cytotoxicity of this defensin on K562 cells could be related to apoptosis mediated by these molecules (Figure 7). In support of the above, the block of calpain activity with the specific inhibitor N-Acetyl-Leu-Leu-norleucinal decreased the apoptosis induced by γ-thionin in K562 cells (~19%) (Figure 8).

### 2.4. γ-Thionin Induces Global Epigenetic Modifications on Histone 3 in the K562 Cells

**Figure 9:**
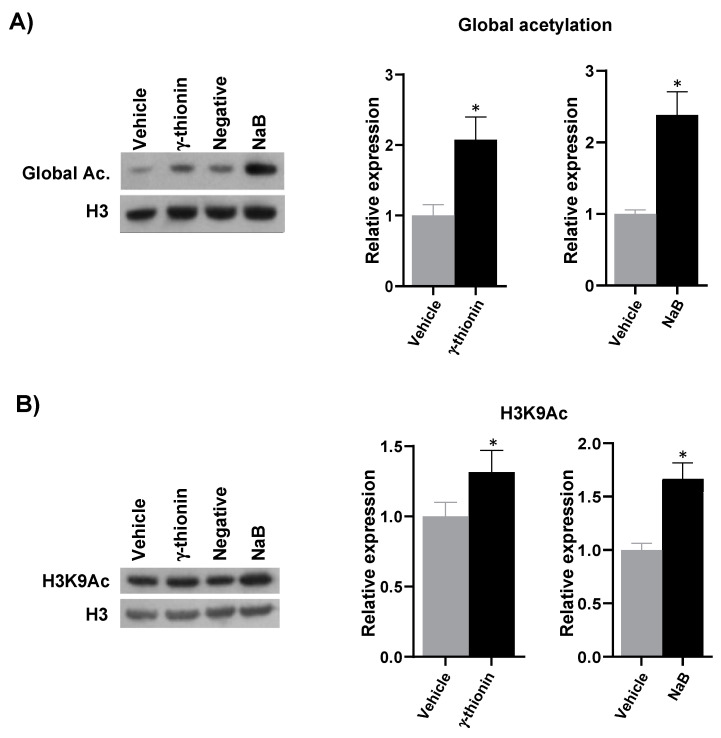
γ-thionin increases histone 3 acetylation in K562 cells. (**A**) Global acetylation marks (H3K9, K14, K18, K23, K27) and (**B**) Acetylation marks of lysine 9 (H3K9Ac) were assessed. A representative Western blot image is shown in each section. Histones were extracted from cells treated for 24 h with γ-thionin IC_50_, vehicle, or NaB. Proteins were separated by SDS-PAGE, and Western transfer assays were performed. Densitometric analysis plots corresponding to Western blot are shown. Histone 3 levels were used as a loading control. Values of γ-thionin were normalized to the vehicle (DMSO), and NaB values were normalized to untreated cells (negative). Data were analyzed by the *t*-student test concerning the vehicle. * Indicates statistically significant differences concerning the vehicle. *n* = 3. Values represent the mean ± SE of three independent experiments. γ-thionin = IC_50_ (290 µg/mL); vehicle = DMSO 1.2%; NaB: 3.5 mM.

We analyzed whether the cytotoxic effect of γ-thionin on the leukemia K562 cell line was related to epigenetic modifications. The following epigenetic marks on histone 3 were analyzed: global acetylation (H3K9, K14, K18, K23, K27), lysine 9 acetylation (H3K9Ac), lysine 9 trimethylation (H3K9me3), lysine 9 dimethylation (H3K9me2), lysine 4 trimethylation (H3K4me3), and serine 10 phosphorylation (H3S10P). The results showed that γ-thionin up-regulates (~2-fold) the global H3 acetylation (Figure 9A) and the H3K9Ac marks (~1.5-fold) (Figure 9B). NaB was used as a positive control for acetylation marks in both cases.

**Figure 10:**
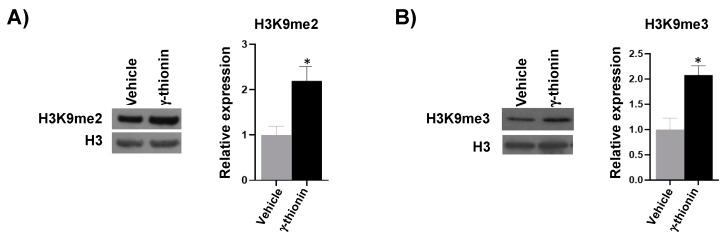
γ-thionin increases histone 3 methylation in K562 cells. (**A**) Lysine 9 dimethylation (H3K9me2) and (**B**) Lysine 9 trimethylation marks (H3K9me3) were assessed. A representative Western blot image is shown in each section. Histones were extracted from cells treated for 24 h with γ-thionin IC_50_ or vehicle. Proteins were separated by SDS-PAGE, and Western assays were performed. Densitometric analysis plots corresponding to Western blot are shown. H3 levels were used as a loading control. Values of γ-thionin were normalized to the vehicle (DMSO). Data were analyzed by the *t*-student test concerning the vehicle. * Indicates statistically significant differences concerning the vehicle. *n* = 3. Values represent the mean ± SE of three independent experiments. γ-thionin = IC_50_ (290 µg/mL); vehicle = DMSO 1.2%.

In addition, γ-thionin increased histone 3 lysine 9 (H3K9me) methylation marks. Furthermore, it up-regulated ~2-fold dimethylation and trimethylation marks (Figure 10A and B, respectively) in the K562 cells. Additionally, two additional epigenetic marks on histone 3 were analyzed: trimethylation of lysine K4 (H3K4me3) and phosphorylation of serine 10 (H3S10P). However, the treatment with γ-thionin did not modify these marks.

## 3. Discussion

In developing alternative therapies against cancer, plant antimicrobial peptides have attracted attention for their cytotoxicity, efficacy, and selectivity against some types of cancer [2,3]. Hence, we showed that the plant defensin γ-thionin (*Capsicum chinense*) is cytotoxic against K562 leukemia cells and that its mechanism of action is by caspase-independent apoptosis mediated by calpains. In addition, γ-thionin regulates histone epigenetic marks.

γ-thionin was cytotoxic to K562 cells in a concentration-dependent manner, with an IC_50_ = 290 μg/mL (50.26 μM) (Figure 1). Interestingly, the γ-thionin IC_50_ was higher concerning the effect of other plant defensins on leukemia cells, such as NAD1 (2.4 μM), coccinin (30 μM), phaseococcin (40 μM) and gymnin (50 μM) [28,29,30]. In addition, γ-thionin defensin was not cytotoxic against human peripheral blood mononuclear cells, as previously reported [26], suggesting that it could be selective against cancer cells; however, more experiments are needed to prove it. In addition, it would be important to evaluate whether γ-thionin defensin can act synergistically with cytostatic agents, e.g., doxorubicin, as reported for avocado defensin PaDef [24]. This could reduce the side effects of conventional treatments and expand the therapeutic possibilities of AMPs. Finally, it would be interesting to analyze the possibility to target cancer cells using this peptide in combination with nanoparticles.

The primary mechanism of cytotoxicity reported for plant defensins on leukemia cells involves membrane damage; this is the case for defensin NaD1 (*N. alata*) and TPP3 (*L. esculentum*) [13,14]. However, γ-thionin did not affect the membrane integrity of K562 cells (Figure 2), which suggests a different mechanism for this peptide. Notably, the cell morphology was affected, showing structures like-apoptotic bodies (Figure 1D); an observation that was corroborated by flow cytometry (Figure 3).

Searching for molecules that activate apoptosis in cancer cells and whose effects are related to modifications in chromatin remodeling could be an attractive anticancer strategy. Apoptosis is a process of cell death that occurs through two principal mechanisms, the intrinsic and extrinsic pathways, and is a cytotoxic mechanism described for plant defensins. For example, radish RsAFP2 and *Heuchera sanguinea* HsAFP1 defensins induce apoptosis in *Candida albicans* [31,32]. In concordance, we demonstrated that avocado PaDef defensin activates caspase-dependent apoptosis in MCF-7 and K562 cells [15,16]. Interestingly, this study provides evidence that γ-thionin cytotoxicity on K562 cells occurs through caspase-independent apoptosis because the activity of caspases 8 and 9 was not detected (Figure 4). In agreement, the mitochondrial membrane potential (ΔΨm) and the intracellular calcium release of K562 cells were modified by γ-thionin at short intervals (Figure 5 and Figure 6); both phenomena are characteristic of caspase-independent apoptosis mediated by calpains [18,19,20,21]. Accordingly, the activity of calpains was detected after 1 h of treatment (Figure 7). Similarly, the human cathelicidin LL-37 activates calpain-dependent apoptosis in Jurkat T leukemia cells [22]. In addition, the antimicrobial peptide epinecidin-1 (epi-1) from the orange-spotted grouper fish (*Epinephelus coioides*) activates caspase-independent cell death in synovial sarcoma cells SW982 cells, increased intracellular calcium levels, ROS production, and calpain activity [33]. However, both AMPs are not defensins, LL-37 is a cathelicidin, and epi-1 is an α-helix peptide. This is the first report of defensin-inducing caspase-independent apoptosis mediated by calpains in leukemia cells.

Epigenetic alterations in histones are essential in developing and maintaining diseases such as cancer. The recovery of altered cellular phenotypes through epigenetic modifications is possible because, unlike genetic determinants, epigenetic alterations are reversible, which makes the search for molecules with epigenetic activity in cancer attractive as a treatment alternative for these diseases. For this reason, we analyzed whether the concentration at which γ-thionin triggers K562 cell death induces epigenetic modifications, as reported for PaDef defensin in the Jurkat leukemia cell line [24]. The results indicate that γ-thionin increased acetylation and methylation marks on histone 3 in K562 cells (Figure 9 and Figure 10). The increase in acetylation marks could result from inhibiting histone deacetylases (HDACs). This agrees with a previous report showing that γ-thionin inhibits the HDACs activity in MCF-7 breast cancer cells and whose inhibition increases the acetylation mark of lysine 9 on histone 3 (H3K9Ac) [34]. In addition, the inhibitory HDACs activity of γ-thionin could explain why calpain blockade decreased apoptotic activity (Figure 8) but did not completely block it. Several mechanisms by which inhibitors of HDACs (iHDACs) trigger apoptosis have been described [35]. Some are caspase-independent; for example, suberoylanilide hydroxamic acid (SAHA) induces apoptosis through a mitochondrial pathway in which caspases are not involved [36]. However, further experiments to evaluate whether γ-thionin affects the HDAC activity in K562 cells are necessary to clarify this point. Regarding the increase in methylation marks, it is necessary to evaluate the activity of either histone methyl transferases or histone methyl transferase inhibitors, which could give us a broader picture of the γ-thionin effects on K562 cells. On the other hand, it has been documented that iHDACs can inhibit demethylase enzymes, such is the case of the iHDACs AR42 and MS-275 that transcriptionally repress some members of the JARID1 family, in charge of the demethylation of histone 3 at lysine 4 (H3K4me) with a consequent increase in the methylation mark [36]. This could give a guideline on the duality of γ-thionin modified marks; however, as mentioned above, evaluating γ-thionin on HDAC activity is necessary. In conclusion, the results showed in this work indicate that γ-thionin activates caspase-independent apoptosis via calpains in K562 cells and regulates epigenetic marks.

## 4. Materials and Methods

### 4.1. Peptide

The antimicrobial peptide γ-thionin used in this work corresponds to the mature region (QNNICKTTSKHFKGLCFADSKCRKVCIQEDKFEDGHCSKLQRKCLCTKNC, 50 aa) (Genbank KC007441), which was chemically synthesized and obtained from BIOMATIK (purity > 90%).

### 4.2. Mammalian Cell Culture

The human leukemia cell line K562 was obtained from American Type Culture Collection. Cells were routinely cultured in RPMI-1640 Media (Sigma-Aldrich, St. Louis, MO, USA) supplemented with 10% (*v*/*v*) fetal bovine serum (Corning, NY, USA) and 100 U/mL of penicillin and streptomycin (Gibco, Waltham, MA, USA) and grown in an atmosphere of 5% CO_2_ at 37 °C, as reported [16]. In addition, Human Peripheral Blood Mononuclear Cells (PBMC) were obtained from the blood of healthy men volunteers. The PBMC were isolated by density gradient centrifugation using Ficoll-Paque ^Tm^ Plus (GE Healthcare, Chicago, IL, USA), and the cells were cultured under the same conditions mentioned above.

### 4.3. Cell Viability Assays

The MTT [3-(4,5-dimethylthiazol-2-yl)-2,5 diphenyl tetrazolium bromide] assay was used to assess the cytotoxicity of γ-thionin [16]. Briefly, K562 cells were synchronized in the RPMI-1640 medium without serum (12 h). Cells were seeded in a 96-well plate at a density of 2 × 10^4^ cells/well and cultured with γ-thionin peptide at various concentrations (10, 25, 50, 100, 200, and 300 μg/mL). After 24 h of incubation, 10 μL of MTT solution (5 mg/mL, Sigma-Aldrich, St. Louis, MO, USA) was added to each well, and plates were incubated at 37 °C in 5% CO_2_ for 4 h. Then, 100 µL of isopropyl alcohol: HCl (19:1) was added to dissolve formazan crystals and was incubated by 20 min. Absorbance was measured at 595 nm using a microplate reader (iMark Microplate Absorbance Reader, BioRad, CA, USA). Actinomycin D was used as cell death-positive control. The results were reported as the percentage of viability concerning vehicle (DMSO 1.2%). Using Excel (Microsoft), the half maximal inhibitory concentration (IC_50_ = 290 μg/mL) was determined by regression analysis. Additionally, SYTO^®®^ 9 green-fluorescent nucleic acid stain and propidium iodide was used to validate IC_50_ by flow cytometry, according to the manufacturer’s instructions. K562 cells were prepared as previously described and treated for 24 h with IC_50_. The measurement was carried out using a BD Accuri™ C6 flow cytometer (BD Biosciences, San Jose, CA, USA). IC50 concentration of γ-thionin was used for the rest of the experiments.

### 4.4. Measurement of the Transmembrane Potential

The cell transmembrane potential depolarization was measured using the membrane potential sensitive dye 3,3′-dipropylthiadicarbocyanine iodide, DiSC3 (5) (Sigma-Aldrich, St. Louis, MO, USA) [15]. For this, K562 cells were cultured in RPMI-1640 Media (Sigma) supplemented with 10% (*v*/*v*) fetal bovine serum (Corning) and 100 U/mL penicillin and streptomycin (Gibco, Waltham, MA, USA). Cells were grown in an atmosphere of 5% CO_2_ at 37 °C for 24 h. Then, K562 cells (1 × 10^5^ cells/well) seeded in 96-well black-wall plates were incubated with 0.2 mM DiSC3 (5) (dissolved in Hanks’ HEPES buffer) for 30 min in a CO_2_ incubator. The fluorescence intensity was monitored for 2 h in a Varioskan spectrophotometer (Thermo Scientific, Waltham, MA, USA). DMSO 5% (Sigma-Aldrich, St. Louis, MO, USA) was used as a positive control.

### 4.5. Apoptosis and Caspases Activation

The apoptosis rate was assessed using a BD Accuri™ C6 flow cytometer (BD Biosciences, San Jose, CA, USA) employing Annexin V (Annexin V, Alexa Fluor 488 conjugate, Invitrogen, Carlsbad, CA, USA) and 7AAD (Bio Legend, San Diego, CA, USA) according to the manufacturer’s instructions [15]. A total of 10,000 events were collected. The data were analyzed using FlowJo software version 10.4 (TreeStar Inc., Ashland, OR, USA). Actinomycin D (Sigma-Aldrich, 80 μg/mL) was used as a positive control.

The activation of caspases 8 and 9 was evaluated with Caspase-Glo 8 and 9 kits (Promega, Madison, WI, USA) according to the manufacturer’s instructions [16]. K562 cells (6 × 10^4^/ well) were seeded in white 96-well plates and incubated with IC_50_ or vehicle in the serum-free medium for 12 or 24 h. The luminescence was detected using a Varioskan spectrophotometer (Thermo Scientific). Actinomycin D (Sigma-Aldrich, 80 μg/mL) was used as a positive control.

### 4.6. Evaluation of Mitochondrial Membrane Potential (ΔΨm)

The changes in mitochondrial membrane potential were monitored using the JC-1 dye (BD Biosciences) in a BD Accuri™ C6 flow cytometer (BD Biosciences) [15]. The K562 cells (1 × 10^5^ cells/well) were cultured in 96-well plates and treated with γ-thionin IC_50_ or vehicle for 6, 12, and 24 h. The cells were treated according to the manufacturer’s instructions. The fluorescence was measured in a BD Accuri ™ C6 flow cytometer (BD Biosciences). The data were analyzed using FlowJo software version 10.4 (TreeStar, Inc., San Carlos, CA, USA). Actinomycin D (Sigma-Aldrich, 80 μg/mL) was used as a positive control.

### 4.7. Calcium Efflux Testing

Calcium efflux was measured by flow cytometry in a BD Accuri™ C6 flow cytometer (BD Biosciences) using a Calcium Assay Kit (BD Biosciences) according to the manufacturer’s instructions [15]. The K562 cells (1 × 10^5^ cells/ well) seeded in 96-well plates were incubated with the indicator dye for 1 h. A baseline fluorescence (3 min) was established and treatments were added (γ-thionin IC_50_ or vehicle). The fluorescence intensity was monitored by flow cytometry without interruption for another 3 min. The data were analyzed with Excel (Microsoft). The Phorbol Myristate Acetate (3 μM; PMA, Sigma-Aldrich) was used as a positive control.

### 4.8. Activation of Calpains

The activation of calpains was assessed with the Calpain-Glo kit (Promega, Madison, WI, USA) according to the manufacturer’s instructions. Briefly, K562 cells (6 × 10^4^/ well) were seeded in white 96-well plates and incubated with IC_50_ or vehicle in serum-free medium for 1, 2, 4, 12, and 24 h. The luminescence data were collected in a Varioskan spectrophotometer (Thermo Scientific). K562 cells were incubated with the calpains inhibitor N-Acetyl-Leu-Leu-norleucinal (5 mg/mL) for 1 h and then treated with γ-thionin IC_50_ by 24 h, and apoptosis rate was determined as described above. The cisplatin (1 μg/mL, Pisa) was used as a positive control.

### 4.9. Histone Extraction and Western Blot Analysis

Cells (1 × 10^6^ cells) were synchronized for 18 h by serum deprivation, then treated with γ-thionin IC_50_ and vehicle (DMSO 1.16%) for 24 h. Then, histone extraction was carried out by acid extraction, as reported [37]. Briefly, the cells were resuspended in an H-lysis solution (0.25 M sucrose, 3 mM CaCl_2_, 1 mM Tris pH 8, and 0.5% NP-40). Subsequently, the cells were recovered and washed with an H-wash solution (300 mM NaCl, 5 mM MgCl_2_, 5 mM DTT and 0.5% NP-40). Histone extraction was accomplished with an H-extraction solution (0.5 M HCl, 10% glycerol, and 0.1 M 2-mercaptoethylamine-HCl). Then, the supernatant was recovered by centrifugation (13,000 rpm for 10 min at 4 °C), and cold acetone was added at a 1:5 ratio. Finally, histones were precipitated for 5 days under refrigeration. The precipitate was recovered by centrifugation (13,000 rpm for 10 min), dissolved in sterile deionized water (20 μL), and stored at −80 °C until use.

For western blot analysis, the histones were separated in a 15% SDS-PAGE gel and transferred to a PVDF membrane by semi-dry transfer, as reported [37]. The membranes were blocked with 5% nonfat dry milk in cold PBS at 4 °C overnight. Furthermore, the membranes were washed three times with cold TBS, and the primary antibody (1:1000) was added and incubated at 4 °C overnight. The following antibodies were used to evaluate: global acetylation (H3K9, K14, K18, K23, K27) (Abcam ab47915, Boston, MA, USA), H3K9ac (Abcam, ab10812) for acetylation, H3K9me2 (Abcam Ab1220), and H3K9me3 (Abcam, ab8898) for di- and tri-methylation, respectively. In addition, the antibody for histone H3 (Abcam, ab1791) was used for loading control. Furthermore, the membranes were incubated with horseradish peroxidase-coupled anti-IgG secondary antibody (1:3000) (Cell Signaling Technology, Danvers, MA, USA). The membranes were washed three times and revealed with Western ECL. The intensity of the signals was quantified by densitometry using the ImageJ software. Sodium butyrate (3.5 mM) was used as a positive control for H3K9 acetylation induction and negative for H3K9me2/3 methylation induction. Data were normalized concerning histone H3 and shown as the relative level of expression concerning the vehicle.

### 4.10. Statistical Analysis

The data were obtained from three independent experiments performed in triplicate. The significance of the differences was assessed using Student’s *t*-test with PRISM 8.02. software. A *p* < 0.05 was considered significant.

## 5. Conclusions

The plant defensin γ-thionin triggers caspase-independent apoptosis via calpains in K562 cells and regulates epigenetic marks, a novel property of this plant defensin.

## Data Availability

The raw data supporting the conclusions of this article will be made available by the authors, without undue reservation, to any qualified researcher.

## Sample Availability

Samples of the compounds are not available from the authors.
